# Reinfection and post-vaccination infection by SARS-CoV-2 in nursing professionals: a prospective cohort study, Recife, 2020-2023

**DOI:** 10.1590/S2237-96222025v34e20240162.en

**Published:** 2025-09-08

**Authors:** Thaís Milena da Silva Mesquita Verissimo, Ulisses Ramos Montarroyos, Demetrius Montenegro, Cláudio Luiz França, Carolline Araújo Mariz

**Affiliations:** 1 Universidade de Pernambuco, Faculdade de Ciências Médicas, Recife, PE, Brazil; 2 Instituto Aggeu Magalhães, Fundação Oswaldo Cruz, Recife, PE, Brazil

**Keywords:** Reinfection, SARS-CoV-2, COVID-19 Vaccines, Nursing, Cohort Studies, Reinfección, SARS-CoV-2, Vacunas contra la COVID-19, Enfermería, Estudios de Cohortes

## Abstract

**Objective:**

To estimate the incidence and factors associated with SARS-CoV-2 reinfection and post-vaccination infection in nursing professionals.

**Methods:**

This was a prospective, descriptive, and analytical cohort study conducted in Recife from March 2020 to January 2023, following 399 nursing professionals. The cumulative incidences of COVID-19 infection and reinfection were estimated with a 95% confidence interval (95%CI).

**Results:**

Among the participants, 71.9% (95%CI 67.3; 76.1) had SARS-CoV-2 infection; of these, 26.6% (95%CI 22.5; 31.1) were reinfected. Regarding the timing of infection, 46.4% (95%CI 41.5; 51.3) of participants were infected before the first dose of the vaccine. After the first dose, the incidence of infection decreased to 25.6% (95%CI 21.5; 30.1), highlighting the importance of vaccination. Nursing technicians were the category with the highest risk of infection. Women were at higher risk of infection and reinfection.

**Conclusion:**

This finding of a reduced occurrence of infections among individuals vaccinated with at least one dose and with a history of prior SARS-CoV-2 infection reinforces the evidence base for hybrid immunity. However, despite the number of infections and reinfections among these professionals, further research is needed to systematize information on primary and recurrent infection cases in this population group, in order to enhance understanding of the phenomenon and improve surveillance processes, as well as prevention, control, and care measures targeting this professional category.

## Resumen

**Objetivo:**

Estimar la incidencia y los factores asociados a la reinfección por SARS-CoV-2 y la infección posvacunal en profesionales de enfermería.

**Métodos:**

Se trata de un estudio de cohorte prospectivo, de carácter descriptivo y analítico, realizado en Recife, de marzo de 2020 a enero de 2023, mediante el seguimiento de 399 profesionales de enfermería. Se estimaron las incidencias acumuladas de infección y reinfección por covid-19, con un intervalo de confianza del 95% (IC95%).

**Resultados:**

Entre los participantes, el 71,9% (IC95% 67,3; 76,1) presentó infección por SARS-CoV-2; de estos, el 26,6% (IC95% 22,5; 31,1) se reinfectó. En cuanto al momento de la infección, el 46,4% (IC95% 41,5; 51,3) de los participantes se infectaron antes de la primera dosis de la vacuna. Tras la primera dosis, la incidencia de la infección se redujo al 25,6% (IC95% 21,5; 30,1), lo que puso de manifiesto la importancia de la inmunización vacunal. Los técnicos de enfermería constituyeron la categoría con mayor riesgo de infección. Las mujeres presentaron un mayor riesgo de infección y reinfección.

**Conclusión:**

Este hallazgo de reducción en la incidencia de infecciones en individuos vacunados con al menos una dosis de la vacuna y con antecedentes de infección previa por SARS-CoV-2 refuerza la base de pruebas sobre la inmunidad híbrida. Sin embargo, a pesar del número de infecciones y reinfecciones de estos profesionales, es necesario avanzar en el desarrollo de investigaciones para sistematizar la información sobre los casos de infección primaria y recurrente en este grupo poblacional, con el fin de comprender mejor el fenómeno y adaptar los procesos de vigilancia, las medidas de prevención, control y atención a esta categoría profesional.

**Ethical aspects**

This research respected ethical principles, having obtained the following approval data:

Research Ethics Committee: Fundação Oswaldo Cruz

Opinion number: 4,021,099

Approval date: 12/5/2020

Certificate of Submission for Ethical Appraisal: 30629220.8.0000.0008

Informed Consent Form: Obtained from all participants prior to data collection.

## Introduction

From March 2020 to May 2023, COVID-19 was declared a pandemic and a global health emergency with substantial public health consequences (1). SARS-CoV-2, a single-stranded RNA virus of the Betacoronavirus genus (2), in addition to being highly contagious, has caused a significant number of deaths (3). As of March 2023, more than 760 million cases had been confirmed and over 6.8 million deaths had been reported worldwide (4).

While the epidemiological profile of certain viral infections may result in lifelong immunity, some types of coronaviruses have been associated with repeated reinfections over time. In New York, the profile of recurrent infections by other endemic coronaviruses (HKU1, 229E, NL63, and OC43) was evaluated, and 14.0% (12/86) of individuals who tested positive experienced multiple reinfections with the same seasonal coronavirus (5). Although SARS-CoV-2 has been present in the human population for more than three years, reinfection cases have also been reported in the scientific literature (6).

In Qatar, a cohort of 43,044 individuals who tested positive for SARS-CoV-2 antibodies was followed for approximately 35 weeks. Through viral genome sequencing, the cumulative risk was estimated at approximately 2 cases per 1,000 individuals, and the reinfection incidence rate at less than 1 case per 10,000 individuals per week (7).

In China (8), a reinfection rate of 14.5% (25/172) was reported using RT-PCR (reverse transcription polymerase chain reaction) for SARS-CoV-2. At Tongji Hospital in Wuhan, a reinfection frequency of 21.4% (15/70) was observed. At Zhongnan Hospital of Wuhan University, the rate was 9.0% (5/55) (9-10).

The duration of protective immunity against SARS-CoV-2 remains a current topic in ongoing research and represents an open question. Observational studies suggest that natural immunity may offer equal or greater protection against SARS-CoV-2 infection compared to individuals who received two doses of a messenger RNA (mRNA) vaccine. The combination of a previous SARS-CoV-2 infection and vaccination, referred to as hybrid immunity, appears to provide greater protection against SARS-CoV-2 infection (11).

In Kentucky, the association between vaccination and SARS-CoV-2 reinfection was evaluated between May and June 2021 in individuals previously infected in 2020. As a result, unvaccinated individuals were twice as likely to be reinfected compared to those who were fully vaccinated (12).

Brazilian studies on SARS-CoV-2 reinfection among healthcare workers are scarce, particularly among nursing professionals—a population that presents specific vulnerabilities due to prolonged exposure through direct care of infected patients. It is of vital importance to develop additional research to systematize information on cases occurring within this population group, in order to generate data to support understanding of the phenomenon and contribute to the improvement of surveillance processes, as well as prevention, control, and care measures for healthcare professionals. 

This study aims to estimate the incidence and associated factors of SARS-CoV-2 reinfection and post-vaccination infection among nurses and nursing technicians. 

## Methods

### Participants

The study population consisted of nurses and nursing technicians who provided care to suspected or confirmed COVID-19 cases during the pandemic in healthcare facilities located in the metropolitan area of Recife. 

### Design

This was a prospective cohort study conducted in the metropolitan area of Recife, as a continuation of the cohort entitled “Assessment of risks for healthcare professionals caring for people with COVID-19,” initiated in March 2020 in the state of Pernambuco. In that cohort, healthcare workers were followed for six months to assess their risk of COVID-19 infection (13). This study continued the follow-up of nursing professionals recruited in the first cohort, from May 2020 (moment of recruitment) to January 2023, with the aim of obtaining data on the occurrence of SARS-CoV-2 reinfection following the introduction of vaccination in the state. 

### Data sources and measurement

The sampling technique used, known as Respondent-Driven Sampling, was applied to select participants for the first cohort. These participants were selected to estimate the incidence of infection with the novel coronavirus (14). This technique was chosen due to limitations on conducting in-person interviews caused by the lockdown during the pandemic and the lack of an official registry of frontline healthcare professionals working in emergency rooms, hospitals, and newly established field hospitals.

Respondent-Driven Sampling was based on snowball sampling to address the problem with randomization. It differed from simple random sampling in that recruitment was carried out through the personal networks of initially selected individuals (referred to as “seeds”), expanding the study population through contact chains. 

To ensure a broad representation of professional categories, five key informants (“seeds”) from different healthcare units were selected. Each seed was allowed to nominate up to five contacts within their professional category. These professionals, in turn, nominated five additional professionals, forming the sample through “waves of contacts.” Recruitment ended either upon saturation of contacts or the expiration of the predetermined data collection period. 

Data collection for the study was divided into two phases of the cohort. The first phase involved the use of data collected in the initial cohort—from May 2020 to February 2021—relating to the exposure of nursing professionals to the risk of COVID-19 infection (biological data, comorbidities, work-related data, and information about SARS-CoV-2 infections acquired during this period). In the second phase, nursing professionals who participated in the first phase were recruited to collect data regarding vaccination, infection, and reinfection with COVID-19 between 6 April, 2020, and 7 January, 2023. A total of 734 participants were recruited during this period and included in the study. Of these, 399 (54.3%) were selected after applying exclusion criteria (professionals with incomplete or insufficient information or those who could not be contacted), and 335 (45.6%) were considered sample loss. 

Participant data were obtained through telephone contact using registration information initially collected during the first cohort phase, and a structured questionnaire was applied to gather information. 

Reinfection was defined as microbiologically diagnosed SARS-CoV-2 cases, confirmed by two positive tests with an interval of more than 90 days between them, in accordance with the diagnostic and testing policy recommended by the World Health Organization at the time of the study. The diagnostic tests considered valid in Brazil during the study period included RT-qPCR molecular tests via nasal or nasopharyngeal swabs, and rapid serological and antigen tests for symptomatic cases. 

### Variables 

The dependent variables were: post-vaccination COVID-19 infection (yes, no) and COVID-19 reinfection (yes, no). The independent variables were: vaccination status at the time of infection or reinfection (unvaccinated, first dose, second dose, third dose); and profession (nurse, nursing technician). 

Covariates collected at the time the nursing professionals entered the cohort were included in the study: age, sex, comorbidities (diabetes, hypertension, obesity, heart disease, kidney disease), workplace (Family Health Program, emergency care unit, public hospital, private hospital), work setting (intensive care unit, outpatient clinic, inpatient ward), and data on the first infection (date of first test performed, type of test [RT-PCR, rapid test, serology], and symptoms presented). 

A descriptive analysis of the nursing professionals’ profile was performed by professional category, sex, age group, comorbidities, and work-related variables. The cumulative incidence of infection and reinfection with COVID-19 and the 95% confidence interval (95%CI) were estimated. To analyze the association of infection and reinfection risks, a multinomial model was used, considering non-infected individuals as the reference group. A multivariate model was applied to adjust the risk of eligible variables. Variables with a significance level below 20.0% in the bivariate analysis were considered eligible for the final model, and those with a significance level below 10.0% remained in the model after multivariate adjustment. 

Considering that the sampling method used in the study involved clusters, to minimize selection bias related to subject independence, a statistical model was applied to adjust for clustering effects when estimating incidence and relative risk. The model used was the generalized linear mixed-effects model. 

## Results

Between 6 April, 2020, and 7 January, 2023, a total of 734 nursing professionals were initially recruited and included in the study. However, 335 of them either had insufficient information or could not be contacted. Thus, 399 professionals were selected for inclusion in the analysis. 

The majority of participants were female (82.2%) with a median age of 37 years; 38.1% of participants had at least one comorbidity, with overweight/obesity (14.8%) and hypertension (14.8%) being the most prevalent (Table 1). 

**Table 1:** Frequency distribution of characteristics of nurses and nursing technicians who provided care to suspected or confirmed COVID-19 case Metropolitan Region of Recife, 2020-2023

Variables	n=399 (%)
**Sex**	
Male	47 (11.8)
Female	352 (88.2)
Age (mean ± SD^a^)	37.6 ± 9.3
**Occupation**	
Nurse	259 (64.9)
Nursing technician	140 (34.1)
Comorbidities^b^	152 (38.1)
Diabetes	9 (2.3)
Hypertension	59 (14.8)
Obesity	59 (14.8)
Heart diseases	7 (1.7)
Kidney disease	1 (0.2)
**Region o^f^ workb**	
Recife	263 (65.9)
Metropolitan region	175 (43.9)
Countryside	14 (3.5)
**Number of concurrent jobs**	
1	139 (34.8)
2	223 (55.9)
≥3	37 (9.3)
**Work sector**	
Outpatient clinic/inpatient ward	64 (16.0)
Intensive care unit/emergency department	212 (53.1)
Another sector	123 (30.8)
Public sector	324 (81.2)
Private sector	24 (6.0)
Both	51( 12.8)

^a^SD: standard deviation; ^b^Non-mutually exclusive categories.

During the cohort period, the incidence of COVID-19 infection among nursing professionals was 71.9% (95%CI 67.3; 76.1); of these, 46.4% occurred before the first vaccine dose, and 25.6% after the first dose. The incidence of reinfection was 26.6% (95%CI 22.5; 31.1). Among the 106 reinfection cases, 79 (74.6%) had one reinfection; 22 (20.7%) had two; and 5 (4.7%) had three reinfections (Table 2). 

**Table 2:** Incidence and 95% confidence interval (95%CI) of infection and reinfection among nurses and nursing technicians who provided care to suspected or confirmed COVID-19 cases. Metropolitan Region of Recife, 2020-2023

Variables	n=399 (%)	(95%CI)
**Infection during the cohort**		
No	112 (28.1)	-
Yes	287 (71.9)	(67.3; 76.1)
Single infection	181 (45.3)	(40.5; 50.3)
Reinfection	106 (26.6)	(22.5; 31.1)
**Timing of primary infection**		
Before the first vaccine dose	185 (46.4)	(41.5; 51.3)
After the first vaccine dose	102 (25.6)	(21.5; 30.1)
**Number of reinfections**		
One	79 (74.6)	(64.4; 87.7)
Two	22 (20.7)	(14.0; 29.6)
Three	5 (4.7)	(2.0; 10.9)

The study examined the association between sociodemographic factors, comorbidities, and occupational variables with the risk of COVID-19 infection and reinfection among nursing professionals. A significant association with sex was observed for both infection and reinfection, as well as a significant association between the number of employment ties and reinfection (Table 3). 

**Table 3:** Multinomial model, with relative risk (RR) and 95% confidence interval (95%CI), for the unadjusted association of factors related to COVID-19 infection and reinfection among nurses and nursing technicians who provided care to suspected or confirmed COVID-19 cases. Metropolitan Region of Recife, 2020–2023

Variables	No infection n=112 (%)	Single infection n=181 (%)	RR (95%CI)	p-value	Reinfection n=106 (%)	RR (95%CI)	p-value
**Sex**							
Male	23 (48.4)	17 (36.2)	Reference	–	7 (14.9)	Reference	–
Female	89 (25.3)	164 (46.6)	2.49 (1.27; 4.91)	0.008	99 (28.1)	3.65 (1.49; 8.92)	0.004
Age (mean ± SD^a^)	36.8 ± 9.7	37.4 ± 9.3	1.01(0.98; 1.03)	0.621	38.6 ± 8.6	1.02 (0.99; 1.05)	0.157
**Occupation**							
Nurse	70 (27.0)	113 (43.6)	Reference	–	76 (29.3)	Reference	–
Nursing technician	42 (30.0)	68 (48.6)	1.00 (0.85; 1.18)	0.991	30 (21.4)	0.87 (0.72; 1.05)	0.150
**Comorbidities**							
No	76 (30.8)	110 (44.5)	Reference	–	61 (24.7)	Reference	–
Yes	36 (23.7)	71 (46.7)	1.36 (0.83; 2.23)	0.222	45 (29.6)	1.56 (0.90; 2.71)	0.116
**Hypertension**							
No	92 (27.1)	154 (45.3)	Reference	–	94 (27.6)	Reference	–
Yes	20 (33.9)	27 (45.8)	0.80 (0.43; 1.52)	0.506	12 (20.3)	0.59 (0.27; 1.27)	0.176
**Obesity**							
No	97 (28.5)	152 (44.7)	Reference	–	91 (26.8)	Reference	–
Yes	15 (25.4)	29 (48.1)	1.24 (0.63; 2.42)	0.541	15 (25.4)	1.07 (0.49; 2.30)	0.871
**Region of work**							
Capital only	61 (28.5)	90 (42.1)	Reference	–	63 (29.4)	Reference	–
Outside the capital only	41 (30.4)	65 (48.2)	1.07 (0.64; 1.78)	0.782	29 (21.5)	0.68 (0.38; 1.24)	0.210
Both in and outside the capital	9 (18.4)	26 (53.1)	1.96 (0.86; 4.47)	0.110	14 (28.6)	1.51 (0.61; 3.74)	0.377
**Number of concurrent jobs**							
One	49 (32.3)	63 (45.3)	Reference	–	27 (19.4)	Reference	–
More than one	63 (24.2)	118 (45.4)	1.46 (0.90; 2.36)	0.127	79 (30.4)	2.28 (1.28; 4.04)	0.005
**Work Sector**							
Outpatient clinic/inpatient ward	22 (34.4)	30 (46.9)	Reference	–	12 (18.7)	Reference	–
Intensive care unit/emergency department	59 (27.8)	94 (44.3)	1.17 (0.62; 2.21)	0.633	59 (27.8)	1.83 (0.83; 4.04)	0.133
Another sector	31 (25.2)	57 (46.3)	1.35 (0.67; 2.72)	0.405	35 (28.5)	2.07 (0.88; 4.86)	0.095
**Type of employing institution**							
Public sector	88 (27.2)	148 (45.7)	Reference	–	88 (27.2)	Reference	–
Private sector	8 (33.3)	10 (41.7)	0.74 (0.28; 1.95)	0.547	6 (25.0)	0.75 (0.25; 2.25)	0.608
Both	16 (31.4)	23 (45.1)	0.85 (0.43; 1.70)	0.656	12 (23.5)	0.75 (0.33; 1.68)	0.483

^a^SD: standard deviation.

After multivariate analysis, sex remained independently associated with both infection and reinfection, with a 2.59-fold increased risk of infection (p-value 0.006) and a 3.96-fold increased risk of reinfection (p-value 0.003) among women compared to men (Table 4). 

**Table 4:** Multivariate analysis, with relative risk (RR) and 95% confidence interval (95%CI), of factors associated with COVID-19 infection and reinfection among nurses and nursing technicians who provided care to suspected or confirmed COVID-19 cases. Metropolitan Region of Recife, 2020–2023

Variables	Primary infection Adjusted RR (95%CI)	p-value	Reinfection Adjusted RR (95%CI)	p-value
**Sex**				
Male	Reference	–	Reference	–
Female	2.59 (1.31; 5.12)	0.006	3.96 (1.60; 9.78)	0.003
**Number of concurrent jobs**				
One	Reference	–	Reference	–
More than one	1.54 (0.93; 2.53)	0.090	2.61 (1.44; 4.72)	0.002
**Occupation**				
Nursing technician	Reference	–	Reference	–
Nurse	0.97 (0.82; 1.14)	0.691	0.82 (0.67; 1.00)	0.046

There was a borderline association between infection and having multiple job ties (p-value 0.090), while the risk of reinfection was significantly higher—2.61 times greater—for this group. Nurses had a lower chance of reinfection compared to nursing technicians (RR 0.82; 95%CI 0.67; 1.00). 

## Discussion

Over the three-year follow-up period, the estimated incidence of SARS-CoV-2 infection among nursing professionals was 71.9% (95%CI 67.3; 76.1), and the incidence of reinfection was 26.6% (95%CI 22.5; 31.1). These findings are consistent with other studies from 2020 and 2021 (15,16), which demonstrate a significant reduction in reinfection risk due to immunity acquired through primary infection, also resulting in a reduced risk of hospitalization and death (16). 

In the United States, a seroprevalence-based cohort study with 3.2 million participants showed a 90.0% reduction (95%CI 81.0; 95.0) in SARS-CoV-2 infection risk when comparing individuals with a positive versus negative antibody test (17). A similar result was found in a prospective cohort of 25,611 participants in England, showing that individuals with prior SARS-CoV-2 infection had an 84.0% lower risk of reinfection over a seven-month follow-up period, compared to those without previous infection (16).

Regarding the timing of infection, this study showed that 46.4% (95%CI 41.5; 51.3) of infections occurred before the first vaccine dose. After the first dose, the frequency of primary infection was 25.6% (95%CI 21.5; 30.1), reflecting a 45.0% reduction in symptomatic cases, thus partially demonstrating the effect of vaccination. At a tertiary hospital in India, individuals who had received two doses of the ChAdOx1 (AstraZeneca) vaccine showed 28.0% effectiveness (95%CI 10.0; 41.0) in preventing symptomatic COVID-19 cases compared to unvaccinated individuals (18).

Some observational studies suggest that natural immunity may provide equal or greater protection against SARS-CoV-2 infection than that conferred by two doses of an mRNA vaccine, although the evidence is not yet conclusive. It has been suggested that the combination of prior SARS-CoV-2 infection and subsequent vaccination, known as hybrid immunity, may provide greater protection against reinfection (11). In line with this, a prospective cohort of asymptomatic healthcare professionals in the United Kingdom found that immunity acquired through infection waned after one year among unvaccinated participants, but remained consistently above 90.0% among those subsequently vaccinated—even in individuals infected more than 18 months earlier (19).

It is important to note that many of the infections and reinfections described in this study occurred during the resurgence of the pandemic caused by the spread of the Omicron variant, which showed two to three times higher transmissibility than the Delta variant (20-21). The Omicron variant has been associated with a substantial ability to evade immunity acquired through prior infection or even vaccine-induced immunity, in contrast with the Beta or Delta variants. This may explain the reinfection cases observed in this study despite prior vaccination. 

With regard to covariates collected at the start of the cohort, female sex was associated with a higher risk of both infection and reinfection. This may be related to greater exposure among women who, in the Brazilian socioeconomic context, are often responsible not only for professional duties but also for caring for children and managing the household (22). In Germany, a higher infection risk was also observed among women, with infection rates greater among women aged 15-59 years. Another possible explanation for female predominance in infection cases is the greater tendency among women to seek healthcare services, which leads to higher testing rates and, consequently, greater case reporting, unlike men who often underestimate health risks (23). At a university hospital in Sergipe, Brazil, 78.8% (95%CI 47.0; 99.8) of recurrent cases occurred among women, with the time interval between the first and second infection ranging from 18 to 134 days (24). In general, the relationship between COVID-19 and its complications in relation to gender is complex and involves differences in comorbidities, behavioral factors, workplace, lifestyles, and biological differences (differences in immune response due to hormonal differences) that need to be further investigated (25). 

In this study, an association was observed between the number of employment ties and increased reinfection risk, with a 2.6-fold higher risk among professionals with more than one job. The work routine of nursing professionals in Brazil has long been characterized by excessive workloads due to the undervaluation of this professional category. Given that this is a predominantly female profession, it is likely that many nurses and nursing technicians became the primary breadwinners during the pandemic, especially considering widespread job loss. This may have compelled them to maintain multiple jobs to support their families. This work overload—often exacerbated by inadequate staffing—and interpersonal challenges in the workplace should be considered determining factors in the illness of this professional group (22).

Nursing technicians had a higher risk of reinfection compared to nurses. It is well known that healthcare professionals—especially those on the front lines of COVID-19 care—are at the highest risk of infection. Nurses and nursing technicians, in particular, perform tasks involving direct and prolonged contact with patients (26). Since August 2020, nursing has been the professional category most affected by COVID-19 in Brazil, with the highest number of confirmed cases among nursing technicians: 88,358 (34.4%) cases among technicians/assistants; 37,366 (14.5%) among nurses; and 27,423 (10.7%) among physicians (27). In northeastern Italy, a significantly higher risk of both primary and recurrent SARS-CoV-2 infection was also observed among nurses and other healthcare workers in direct contact with patients (28). 

Among healthcare professionals, nursing technicians/assistants were among the most vulnerable to SARS-CoV-2 infection and its complications (13). Possible reasons for the high rate of primary infections and reinfections among nursing technicians include closer and longer contact with patients through bedside care, such as administering medication and bathing, performing higher-risk procedures such as tracheal secretion suctioning, and being the first responders in cases of patient complications (29).

This finding of reduced infection rates among individuals vaccinated with at least two doses and with a history of prior SARS-CoV-2 infection reinforces the growing body of evidence on hybrid immunity. Regarding the risk of SARS-CoV-2 infection among nursing professionals, nursing technicians—due to their more frequent and direct contact with patients—were the group at greatest risk of infection. There is a pressing need for further research to systematize information on primary and recurrent infections in this population group to better understand the phenomenon and improve surveillance processes, as well as prevention, control, and care strategies for this professional category.

A limiting factor of this study was the considerable reduction in the sample size of nursing professionals recruited from the initial cohort preceding this study, with the loss of 335 professionals. This may have resulted from difficulties in maintaining contact with these professionals via smartphone, possibly related to the work overload commonly experienced in this professional category, which may have made them less willing or confident to continue participating in the study. This loss was regrettable, as it hindered the ability to obtain even more relevant findings for the current context.

Another limitation was the potential recall bias during the interviews. Given the particularly challenging context of the pandemic, it is likely that some of the professionals recruited may have faced difficulties in providing accurate information during data collection. However, this bias was minimized, as a significant number of participants provided images of their vaccination records.

Another limitation of the study was the use of a non-probabilistic sampling method. Respondent-Driven Sampling was adopted due to the specific conditions imposed by the pandemic: restrictions on conducting face-to-face interviews due to the lockdown, and the absence of a formal registry of frontline healthcare professionals working in emergency rooms, hospitals, and temporary field hospitals. The importance of adopting more robust sampling methods in favourable contexts is worth emphasizing.

It is also important to note the possibility of viral persistence in some of the reinfection cases identified (30). However, this condition was minimized by the criterion adopted in this study, which only considered a new infection when it occurred more than 90 days after the initial infection.

## Data Availability

The dataset and analysis codes used in this study are available at: https://1drv.ms/f/s!AvWBrOBm0qnih5M4Wf3ModiwMav4YQ?e=bHye54.
