# Supplementary material for: Reinfection and post-vaccination infection by SARS-CoV-2 in nursing professionals: a prospective cohort study, Recife, 2020-2023
Source: Epidemiol Serv Saude. 2025 Sep 8;34:e20240162. doi: 10.1590/S2237-96222025v34e20240162.en (PMC12435892; doi:10.1590/S2237-96222025v34e20240162.en)
Supplement: Translation

# Translation: Reinfecção e infecção pós-vacinal por SARS-CoV-2 em profissionais de enfermagem: estudo de coorte prospectiva, Recife, 2020-2023

**DOI:** 10.1590/S2237-96222025v34e20240162.pt

**Authors:** Thaís Milena da Silva Mesquita Verissimo, Ulisses Ramos Montarroyos, Demetrius Montenegro, Cláudio Luiz França, Carolline Araújo Mariz

**Affiliations:** 1 Universidade de Pernambuco, Faculdade de Ciências Médicas, Recife, PE, Brasil; 2 Instituto Aggeu Magalhães, Fundação Oswaldo Cruz, Recife, PE, Brasil

**Keywords:** Reinfecção, SARS-CoV-2, Vacinas Contra Covid-19, Enfermagem, Estudos de Coortes

## Abstract

**Objetivo:**

Estimar a incidência e os fatores associados à reinfecção por SARS-CoV-2 e infecção pós-vacina em profissionais de enfermagem.

**Métodos:**

Tratou-se de estudo de coorte prospectiva, de caráter descritivo e analítico, conduzido no Recife, de março de 2020 a janeiro de 2023, através do acompanhamento de 399 profissionais de enfermagem. Foram estimadas as incidências cumulativas de infecção e reinfecção por covid-19, com intervalo de confiança de 95% (IC95%).

**Resultados:**

Entre os participantes, 71,9% (IC95% 67,3; 76,1) apresentaram infecção por SARS-CoV-2; destes, 26,6% (IC95% 22,5; 31,1) foram reinfectados. Em relação ao momento da ocorrência de infecção, 46,4% (IC95% 41,5; 51,3) dos participantes foram infectados antes da primeira dose da vacina. Após a primeira dose, a incidência de infecção reduziu para 25,6% (IC95% 21,5; 30,1), o que evidenciou a importância da imunização vacinal. Os técnicos de enfermagem constituíram a categoria que apresentou maior risco de infecção. As mulheres apresentaram maior risco de infecção e reinfecção.

**Conclusão:**

Este achado de redução na ocorrência de infecções em indivíduos vacinados com ao menos uma dose da vacina e histórico de infecção prévia por SARS-CoV-2 fortalece a base de evidências acerca da imunidade híbrida. No entanto, a despeito do número de infecções e reinfecções desses profissionais, faz-se necessário o avanço no desenvolvimento de pesquisas a fim de sistematizar informações acerca dos casos de infecção primária e recorrente nesse grupo populacional para melhor compreensão do fenômeno e adequação dos processos de vigilância, medidas de prevenção, controle e atenção a essa categoria profissional.

**Aspectos éticos**

A presente pesquisa respeitou os princípios éticos, obtendo os seguintes dados de aprovação:

Comitê de ética em pesquisa: Fundação Oswaldo Cruz

Número do parecer: 4.021.099

Data de aprovação: 12/5/2020

Certificado de apresentação de apreciação ética: 30629220.8.0000.0008

Registro do consentimento livre e esclarecido: Obtido de todos os participantes antes da coleta.

## Introdução

De março de 2020 a maio de 2023, a covid-19 foi declarada como pandemia e uma emergência de saúde global com consequências substanciais para a saúde pública (1). O SARS-CoV-2, vírus de ácido ribonucleico (RNA) de fita simples, do gênero Betacoronavírus (2), além de ser altamente contagioso, causou elevado número de óbitos (3). Até março de 2023, mais de 760 milhões de casos foram confirmados e mais de 6,8 milhões de mortes foram relatadas globalmente (4).

Embora o quadro epidemiológico de algumas infecções virais resulte em imunidade vitalícia, alguns tipos de coronavírus têm causado reinfecções repetidas ao longo do tempo. Em Nova York, avaliou-se o perfil de infecções recorrentes por outros tipos de coronavírus endêmicos (HKU1, 229E, NL63 e OC43) e verificou-se que 14,0% (12/86) dos indivíduos que testaram positivo tiveram múltiplas reinfecções com o mesmo coronavírus sazonal (5). Apesar de o SARS-CoV-2 estar presente na população humana há mais de três anos, casos de reinfecção também têm sido relatados na literatura científica (6).

No Catar, em uma coorte de 43.044 indivíduos positivos para anticorpos do SARS-CoV-2, acompanhados por cerca de 35 semanas, por meio do sequenciamento do genoma viral, foram estimados o risco cumulativo de, aproximadamente, 2 casos por 1 mil pessoas, e a taxa de incidência de reinfecção de menos de 1 caso por 10 mil pessoas por semana (7).

Na China (8), houve reinfecção de 14,5% (25/172) pelo RT-PCR (reação de transcriptase reversa seguida de reação em cadeia da polimerase) para SARS-CoV-2. No hospital Tongji, em Wuhan, foi observada a frequência de reinfecção de 21,4% (15/70). No hospital Zhongnan da Universidade de Wuhan, a taxa foi de 9,0% (5/55) (9-10).

A duração da imunidade protetora ao SARS-CoV-2 é um tema vigente nas pesquisas atuais e permanece como lacuna a ser respondida. Estudos observacionais indicam que a imunidade natural pode oferecer proteção igual ou maior contra infecções por SARS-CoV-2 em comparação com indivíduos que receberam duas doses de uma vacina de RNA mensageiro (mRNA). A combinação de uma infecção anterior por SARS-CoV-2 e uma respectiva vacinação, denominada imunidade híbrida, parece conferir maior proteção contra infecções por SARS-CoV-2 (11).

Em Kentucky, foi avaliada a associação entre vacinação e reinfecção por SARS-CoV-2 entre maio e junho de 2021 com pessoas previamente infectadas com esse vírus em 2020. Como resultado, as pessoas que não foram vacinadas tiveram duas vezes mais chances de serem reinfectadas em comparação com aquelas com vacinação completa (12).

São escassas as pesquisas brasileiras realizadas sobre reinfecção por SARS-CoV-2 entre profissionais de saúde, principalmente entre profissionais de enfermagem, população que apresenta peculiar vulnerabilidade em decorrência do maior tempo de exposição pelo cuidado direto ao paciente infectado. É de vital importância o desenvolvimento de pesquisas adicionais para sistematizar informações acerca dos casos ocorridos nesse grupo populacional, a fim de obter dados para compreensão do fenômeno e contribuir para a adequação dos processos de vigilância, medidas de prevenção, controle e atenção aos profissionais de saúde. 

Este estudo tem como objetivo estimar a incidência e os fatores associados à reinfecção por SARS-CoV-2 e à infecção pós-vacina em enfermeiros e técnicos de enfermagem. 

## Métodos

### Participantes

A população do estudo foi composta por enfermeiros e técnicos de enfermagem que prestaram atendimento aos casos suspeitos ou confirmados com covid-19, durante o contexto pandêmico, em unidades de saúde localizadas na região metropolitana do Recife. 

### Delineamento

Tratou-se de estudo de coorte prospectivo conduzido na região metropolitana do Recife, em continuidade à coorte “Avaliação dos riscos de profissionais de saúde que cuidam de pessoas com covid-19” realizada em março de 2020 em Pernambuco, em que foram acompanhados profissionais de saúde analisando o risco de infecção por covid-19 durante um período de seis meses (13). Este estudo deu prosseguimento ao acompanhamento dos profissionais de enfermagem recrutados da primeira coorte, desde maio de 2020 (momento do recrutamento) até janeiro de 2023, visando à obtenção de informações sobre a ocorrência de reinfecção por SARS-CoV-2 após o início da oferta de vacina no estado. 

### Fontes de dados e mensuração

A técnica de amostragem utilizada, conhecida como Respondent-Driven Sampling, foi aplicada para a seleção dos participantes da primeira coorte, sendo os mesmos selecionados para estimar a incidência de infecção pelo novo coronavírus (14). Essa técnica foi escolhida como uma abordagem de amostragem devido às restrições na condução de entrevistas presenciais em vista do lockdown ocorrido no contexto da pandemia, além da falta de registro do quadro de profissionais de saúde atuantes na linha de frente que atendem em salas de emergência, hospitais e novos hospitais de campanha.

A técnica amostral Respondent-Driven Sampling teve como referência a amostragem do tipo bola de neve para resolver o problema da aleatoriedade amostral. Distinguiu-se das amostras aleatórias simples, pois o recrutamento se fez a partir de uma rede de conhecidos dos indivíduos selecionados inicialmente (denominados como sementes), ampliando a população de estudo por meio das cadeias de contato. 

Visando garantir a abrangência da amostra das categorias profissionais, foram considerados cinco informantes-chave (sementes) de unidades de saúde diferentes. Cada uma das sementes indicou no máximo cinco contatos dentro da categoria profissional ao qual pertencia. Esses profissionais recrutados pelos indicados pelas sementes, por sua vez, indicaram mais cinco profissionais, formando a amostra por “ondas de contatos”. A finalização do recrutamento ocorreu com a saturação dos contatos ou a finalização do período estipulado para coleta. 

A coleta de dados do estudo foi dividida em duas fases da coorte. A primeira fase consistiu na utilização de dados coletados da primeira coorte — realizada entre maio de 2020 e fevereiro de 2021 — relacionados à exposição de profissionais de enfermagem ao risco de infecção pela covid-19 (dados biológicos, comorbidades, dados relacionados ao trabalho e acerca de infecções causadas pelo SARS-CoV-2 adquiridas durante esse período). Na segunda fase, foram recrutados os profissionais de enfermagem participantes da fase anterior, a fim de serem coletados os dados referentes à vacinação, infecção e reinfecção da covid-19, entre 6 de abril de 2020 e 7 de janeiro de 2023. Um total de 734 participantes foram recrutados nesse período e incluídos no estudo. Destes, 399 (54,3%) foram selecionados após aplicação dos critérios de exclusão (profissionais cujas informações estavam incompletas ou insuficientes, e aqueles em que não foi possível estabelecer contato), sendo considerado como perda 335 (45,6%) profissionais. 

Os dados dos participantes foram obtidos por meio de contato telefônico com esses profissionais recrutados, a partir dos dados cadastrais coletados inicialmente na primeira fase da coorte, sendo aplicado questionário para a coleta de informações. 

Foram considerados como reinfecção os casos de SARS-CoV-2 diagnosticados microbiologicamente, confirmados por meio de dois testes positivos com intervalo maior que 90 dias entre eles, de acordo com a política de diagnóstico e teste sob recomendação da Organização Mundial de Saúde no período da pesquisa. Os testes válidos no Brasil durante o período do estudo foram os testes moleculares de RT-qPCR através de coleta por meio de swabs nasais ou nasofaríngeos, testes rápidos sorológicos e de antígenos para casos sintomáticos. 

### Variáveis 

As variáveis dependentes do estudo foram: infecção pós-vacina pela covid-19 (sim, não) e reinfecção pela covid-19 (sim, não). As variáveis independentes foram: condição vacinal no momento da infecção ou reinfecção (não vacinado, primeira dose, segunda dose, terceira dose); e profissão (enfermeiro, técnico de enfermagem). 

As variáveis coletadas na entrada do profissional de enfermagem na coorte foram incluídas no estudo como covariadas: idade, sexo, comorbidades (diabetes, hipertensão, obesidade, cardiopatia, doença renal), local de trabalho (Programa de Saúde da Família, unidade de pronto atendimento, hospital público, hospital privado), setor de trabalho (unidade de terapia intensiva, ambulatório, enfermaria) e dados da primeira infecção (data do primeiro teste realizado, tipo de teste [RT-PCR, teste rápido, sorológico] e sintomas apresentados). 

Foi realizada análise descritiva do perfil dos profissionais de enfermagem por categoria profissional, sexo, faixa etária, comorbidades e variáveis relacionadas ao trabalho. Estimaram-se a incidência cumulativa de infecção e reinfecção por covid-19 e o intervalo de confiança de 95% (IC95%). Na análise de associação do risco de infecção e reinfecção, foi utilizado modelo multinomial, considerando os não infectados como referência. Modelo multivariado foi aplicado para ajuste do risco das variáveis elegíveis. O critério de elegibilidade das variáveis que compuseram o modelo final foi a significância abaixo de 20,0% na análise bivariada, permanecendo no modelo as variáveis com significância abaixo de 10,0% após o ajuste multivariado. 

Levando em consideração que o tipo de amostragem aplicada no estudo tem caráter de conglomerados, para que o viés de seleção relacionado à independência entre sujeitos seja minimizado, um modelo estatístico para ajustes do efeito de agrupamento foi utilizado para estimar a incidência e o risco relativo. O modelo aplicado foi o geral linearizado de efeitos mistos. 

## Resultados

Entre 6 de abril de 2020 e 7 de janeiro de 2023, 734 profissionais de enfermagem foram recrutados, sendo incluídos na pesquisa inicialmente. No entanto, 335 destes apresentaram informações insuficientes ou não foi possível estabelecer contato. Sendo assim, 399 foram selecionados para a pesquisa. 

A maioria dos participantes foi do sexo feminino (82,2%) e com idade mediana de 37 anos; 38,1% dos pesquisados tinham alguma comorbidade, sendo sobrepeso/obesidade (14,8%) e hipertensão (14,8%) as mais prevalentes (Tabela 1). 

**Tabela 1:** Distribuição de frequência das características de enfermeiros e técnicos de enfermagem que prestaram atendimento a casos suspeitos ou confirmados de covid-19. Região metropolitana do Recife, 2020-2023

Variáveis	n=399 (%)
**Sexo**	
Masculino	47 (11,8)
Feminino	352 (88,2)
Idade (média±DP^a^)	37,6±9,3
**Ocupação**	
Enfermeiro	259 (64,9)
Técnico de enfermagem	140 (34,1)
Comorbidades^b^	152 (38,1)
Diabetes	9 (2,3)
Hipertensão	59 (14,8)
Obesidade	59 (14,8)
Cardiopatias	7 (1,7)
Doença renal	1 (0,2)
**Região em que tr^a^balhab**	
Recife	263 (65,9)
Região metropolitana	175 (43,9)
Interior do estado	14 (3,5)
**Número de vínculos**	
1	139 (34,8)
2	223 (55,9)
≥3	37 (9,3)
**Setor de trabalho**	
Ambulatório/enfermaria	64 (16,0)
Unidade de terapia intensiva/emergência	212 (53,1)
Outro setor	123 (30,8)
Pública	324 (81,2)
Privada	24 (6,0)
Ambas	51 (12,8)

^a^DP: desvio-padrão; ^b^Categorias não excludentes.

Durante a coorte, a incidência de infecção por covid-19 entre os profissionais de enfermagem foi 71,9% (IC95% 67,3; 76,1); destes, 46,4% foram antes da primeira dose da vacina, e 25,6%, após a primeira dose. A incidência de reinfecção foi 26,6% (IC95% 22,5; 31,1). Dos 106 casos de reinfecção, 79 (74,6%) apresentaram uma reinfecção; 22 (20,7%), duas; e 5 (4,7%), três reinfecções (Tabela 2). 

**Tabela 2:** Incidência e intervalo de confiança (IC95%) de infecção e reinfecção de enfermeiros e técnicos de enfermagem que prestaram atendimento a casos suspeitos ou confirmados com covid-19. Região metropolitana do Recife, 2020-2023

Variáveis	n=399 (%)	(IC95%)
**Infecção durante a coorte**		
Não	112 (28,1)	–
Sim	287 (71,9)	(67,3; 76,1)
Uma infecção	181 (45,3)	(40,5; 50,3)
Reinfecção	106 (26,6)	(22,5; 31,1)
**Momento da primoinfecção**		
Antes da primeira dose da vacina	185 (46,4)	(41,5; 51,3)
Após a primeira dose da vacina	102 (25,6)	(21,5; 30,1)
**Número de reinfecções**		
Uma	79 (74,6)	(64,4; 87,7)
Duas	22 (20,7)	(14,0; 29,6)
Três	5 (4,7)	(2,0; 10,9)

Apresentou-se a associação dos fatores relacionados sociodemográficos, comorbidades e relacionados à atividade ocupacional em relação ao risco de infecção e reinfecção pela covid-19 entre os profissionais de enfermagem. Observou-se associação significativa com sexo tanto para infecção quanto reinfecção e significância na associação do número de vínculos com reinfecção (Tabela 3). 

**Tabela 3:** Modelo multinomial, com risco relativo (RR) e intervalo de confiança (IC95%), da associação bruta dos fatores associados à infecção e reinfecção pela covid-19 em enfermeiros e técnicos de enfermagem que prestaram atendimento a casos suspeitos ou confirmados de covid-19. Região metropolitana do Recife, 2020-2023

Variáveis	Sem infecção n=112 (%)	Uma infecção n=181 (%)	RR (IC95%)	p-valor	Reinfecção n=106 (%)	RR (IC95%)	p-valor
Sexo							
Masculino	23 (48,4)	17 (36,2)	Referência	–	7 (14,9)	Referência	–
Feminino	89 (25,3)	164 (46,6)	2,49 (1,27; 4,91)	0,008	99 (28,1)	3,65 (1,49; 8,92)	0,004
Idade (média±DP^a^)	36,8±9,7	37,4±9,3	1,01 (0,98; 1,03)	0,621	38,6±8,6	1,02 (0,99; 1,05)	0,157
**Ocupação**							
Enfermeiro	70 (27,0)	113 (43,6)	Referência	–	76 (29,3)	Referência	–
Técnico de enfermagem	42 (30,0)	68 (48,6)	1,00 (0,85; 1,18)	0,991	30 (21,4)	0,87 (0,72; 1,05)	0,150
**Comorbidades**							
Não	76 (30,8)	110 (44,5)	Referência	–	61 (24,7)	Referência	–
Sim	36 (23,7)	71 (46,7)	1,36 (0,83; 2,23)	0,222	45 (29,6)	1,56 (0,90; 2,71)	0,116
**Hipertensão**							
Não	92 (27,1)	154 (45,3)	Referência	–	94 (27,6)	Referência	–
Sim	20 (33,9)	27 (45,8)	0,80 (0,43; 1,52)	0,506	12 (20,3)	0,59 (0,27; 1,27)	0,176
**Obesidade**							
Não	97 (28,5)	152 (44,7)	Referência	–	91 (26,8)	Referência	–
Sim	15 (25,4)	29 (48,1)	1,24 (0,63; 2,42)	0,541	15 (25,4)	1,07 (0,49; 2,30)	0,871
**Região em que trabalha**							
Apenas na capital	61 (28,5)	90 (42,1)	Referência	–	63 (29,4)	Referência	–
Apenas fora da capital	41 (30,4)	65 (48,2)	1,07 (0,64; 1,78)	0,782	29 (21,5)	0,68 (0,38; 1,24)	0,210
Capital e fora da capital	9 (18,4)	26 (53,1)	1,96 (0,86; 4,47)	0,110	14 (28,6)	1,51 (0,61; 3,74)	0,377
**Número de vínculos**							
Um	49 (32,3)	63 (45,3)	Referência	–	27 (19,4)	Referência	–
Mais de um	63 (24,2)	118 (45,4)	1,46 (0,90; 2,36)	0,127	79 (30,4)	2,28 (1,28; 4,04)	0,005
**Setor de trabalho**							
Ambulatório/enfermaria	22 (34,4)	30 (46,9)	Referência	–	12 (18,7)	Referência	–
Unidade de terapia intensiva/emergência	59 (27,8)	94 (44,3)	1,17 (0,62; 2,21)	0,633	59 (27,8)	1,83 (0,83; 4,04)	0,133
Outro setor	31 (25,2)	57 (46,3)	1,35 (0,67; 2,72)	0,405	35 (28,5)	2,07 (0,88; 4,86)	0,095
**Rede de trabalho**							
Pública	88 (27,2)	148 (45,7)	Referência	–	88 (27,2)	Referência	–
Privada	8 (33,3)	10 (41,7)	0,74 (0,28;1,95)	0,547	6 (25,0)	0,75 (0,25; 2,25)	0,608
Ambas	16 (31,4)	23 (45,1)		0,656	12 (23,5)	0,75 (0,33; 1,68)	0,483

^a^DP: desvio-padrão.

Após a análise multivariada, observou-se que o sexo teve associação independente tanto à infecção como à reinfecção, com aumento de 2,59 vezes no risco de infecção (p-valor 0,006) e 3,96 maior risco de reinfecção (p-valor 0,003) entre as mulheres quando comparado aos homens (Tabela 4). 

**Tabela 4:** Análise multivariada, com risco relativo (RR) e intervalo de confiança (IC95%), dos fatores associados à infecção e reinfecção pela covid-19 em enfermeiros e técnicos de enfermagem que prestaram atendimento a casos suspeitos ou confirmados com covid-19. Região metropolitana do Recife, 2020-2023

Variáveis	Primoinfecção RR ajustado (IC95%)	p-valor	Reinfecção RR ajustado (IC95%)	p-valor
**Sexo**				
Masculino	Referência	–	Referência	–
Feminino	2,59 (1,31; 5,12)	0,006	3,96 (1,60; 9,78)	0,003
**Número de vínculos**				
Um	Referência	–	Referência	–
Mais de um	1,54 (0,93; 2,53)	0,090	2,61 (1,44; 4,72)	0,002
**Ocupação**				
Técnico de enfermagem	Referência	–	Referência	–
Enfermeiro	0,97 (0,82; 1,14)	0,691	0,82 (0,67; 1,00)	0,046

Para a infecção, houve associação limítrofe com maior risco para os profissionais com mais de um vínculo (p-valor 0,090) e associação significativa com aumento de 2,61 vezes no risco de reinfecção para essa população. Os enfermeiros tiveram menor chance de reinfecção em comparação ao grupo de técnicos de enfermagem (RR 0,82; IC95% 0,67; 1,00). 

## Discussão

Ao longo de três anos de acompanhamento, a incidência estimada de infecção por SARS-CoV-2 entre os profissionais de enfermagem foi 71,9% (IC95% 67,3; 76,1)), e a incidência de reinfecção, 26,6% (IC95% 22,5; 31,1). Os resultados deste estudo corroboram outros achados de 2020 e 2021 (15,16), ao evidenciar a redução significativa no risco de reinfecção devido à imunidade adquirida decorrente de primoinfecção, o que também resultou na redução do risco de hospitalizações e mortes (16). 

Nos Estados Unidos, uma coorte baseada em dados de soroprevalência incluindo 3,2 milhões de participantes mostrou redução de 90,0% (IC95% 81,0; 95,0) no risco de infecções por SARS-CoV-2 comparando pacientes com teste de anticorpo positivo versus negativo contra SARS-CoV-2 (17). Resultado semelhante foi observado em outra coorte prospectiva de 25.611 ingleses, na qual mostrou que um indivíduo com infecção prévia por SARS-CoV-2 apresentou risco 84,0% menor de reinfecção, em sete meses de acompanhamento, após infecção primária vs indivíduos não infectados (16).

Em relação ao momento da ocorrência de infecção, este trabalho evidenciou que 46,4% (IC95% 41,5; 51,3) das infecções ocorreram antes da primeira dose da vacina, e, após a primeira dose, a frequência de primoinfecção foi 25,6% (IC95% 21,5; 30,1), redução de 45,0% de casos sintomáticos, o que demostrou, em parte, o efeito da imunização vacinal. Em um hospital terciário na Índia, indivíduos vacinados com duas doses da vacina ChAdOx1 (AstraZeneca) apresentaram eficácia na proteção de casos sintomáticos de covid-19 de 28,0% (IC95% 10,0; 41,0) em comparação com pessoas não vacinadas (18).

Alguns estudos observacionais chegaram a indicar que a imunidade natural pode conferir proteção igual ou maior contra infecções por SARS-CoV-2 do que a imunidade induzida por duas doses de uma vacina de mRNA, mas as evidências ainda não são totalmente suficientes. Foi apontado que a combinação de uma infecção anterior por SARS-CoV-2 e uma respectiva vacinação, denominada imunidade híbrida, pode conferir maior proteção contra reinfecções por SARS-CoV-2 (11). Em concordância, uma coorte prospectiva de profissionais de saúde assintomáticos no Reino Unido apontou que a imunidade adquirida pela infecção diminuiu após um ano em participantes não vacinados, mas permaneceu consistentemente superior a 90,0% naqueles que foram vacinados posteriormente, mesmo em pessoas infectadas há mais de 18 meses (19).

É importante salientar que boa parte dos casos de infecção e reinfecção descritos neste estudo ocorreu no período de recrudescimento da pandemia provocado pela disseminação da variante Ômicron, que mostrou transmissibilidade de duas a três vezes maior que a variante Delta (20-21). Foi sugerido que a variante Ômicron está associada à capacidade substancial de escapar da imunidade adquirida de infecção prévia e até mesmo da imunidade induzida por vacina, em contraste com as variantes Beta ou Delta, o que pode justificar neste estudo os casos de reinfecções ocorridas após vacinação. 

No que se refere à associação de algumas covariadas coletadas no início da coorte, o sexo feminino apresentou maior risco de infecção e reinfecção. Isso pode estar relacionado à maior exposição das mulheres que, no contexto socioeconômico do Brasil, além do ambiente de trabalho, é possível que possuam demandas associadas à dupla jornada de cuidar de filhos, além das atividades voltadas à administração do lar (22). Na Alemanha, também houve maior risco de infecção entre as mulheres, com taxas de infecção maiores entre mulheres de 15-59 anos. Outro fator que pode estar associado à predominância feminina é o de que as mulheres geralmente apresentam maior interesse na busca de serviço de saúde, o que leva à maior taxa de testes realizados e, consequentemente, maior notificação da doença, em contraste com os homens que tendem, muitas vezes, a subestimar os riscos inerentes à saúde (23). No hospital universitário de Sergipe, no Brasil, verificou-se que 78,8% (IC95% 47,0; 99,8) dos casos recorrentes ocorrem entre mulheres, com intervalo de tempo entre a primeira e a segunda infecção de 18 a 134 dias (24). De forma geral, a relação entre covid-19 e suas complicações com o gênero é complexa e envolve diferenças nas comorbidades, fatores comportamentais, local de trabalho, estilos de vida e diferenças biológicas (a diferença na resposta imune devido a diferenças hormonais) que precisam ser mais investigadas (25). 

Neste estudo, observou-se associação em relação ao número de vínculos de trabalho, com aumento de 2,6 vezes do risco de reinfecção para os profissionais com mais de um vínculo. O ritmo laboral dos trabalhadores de enfermagem no Brasil, há muito, é caracterizado por carga horária excessiva de trabalho devido à desvalorização salarial dessa categoria. Considerando ser uma profissão majoritariamente composta por mulheres, é possível que muitas enfermeiras e técnicas de enfermagem tenham assumido o sustento do lar, sobretudo no contexto pandêmico, quando muitos empregos foram perdidos, o que as levou à necessidade de manter mais de um vínculo empregatício para prover a renda familiar. Essa sobrecarga de trabalho, cujo dimensionamento de pessoal na maioria das vezes é inadequado, e as dificuldades nas relações interpessoais devem ser consideradas fatores determinantes para o adoecimento dessa categoria profissional (22).

Os técnicos de enfermagem tiveram maior risco de reinfecção em comparação com o grupo de enfermeiros. Sabe-se que profissionais de saúde, sobretudo aqueles que atuam na linha de frente da covid-19, são os mais propensos ao risco de infecção pela doença, e, ainda de forma mais intensa, os enfermeiros e técnicos de enfermagem possuem atribuições de trabalho que envolvem contato direto com o paciente (26). Desde agosto de 2020, a enfermagem foi apontada como a categoria mais atingida pela covid-19 no Brasil e apresentou o registro de maior número de casos entre técnicos de enfermagem, sendo 88.358 (34,4%) casos de infecção entre técnico/auxiliares de enfermagem; 37.366 (14,5%) entre enfermeiros; e 27.423 (10,7%) entre médicos (27). Na região nordeste da Itália, também foi evidenciado risco de infecção primária e recorrente por SARS-CoV-2 significativamente maior para enfermeiros e outras categorias de profissionais de saúde que atuavam diretamente com o paciente (28). 

Uma das maiores frequências de complicações por SARS-CoV-2 entre profissionais de saúde, e entre eles, técnicos/auxiliares de enfermagem, consistiu na categoria mais vulnerável à infecção (13). Entre as possíveis razões para a elevada taxa de infecções primárias e reinfecções em técnicos de enfermagem, destacaram-se o contato mais próximo e mais longo com os pacientes, envolvendo atividades realizadas à beira do leito, como administração de medicamentos e banho dos pacientes no leito, e realização de procedimentos de maior risco, como aspiração de secreções traqueais, além de serem a primeira linha de atendimento em caso de complicações do paciente (29).

Este achado acerca da redução da ocorrência de infecções em indivíduos vacinados com ao menos duas doses da vacina e histórico de infecção prévia por SARS-CoV-2 fortaleceu a base de evidências acumulada ao longo dos anos acerca da imunidade híbrida. Em relação ao risco de infecção por SARS-CoV-2 em profissionais de enfermagem, os técnicos de enfermagem, por possuírem atribuições ainda mais constantes de contato direto ao paciente, constituíram a categoria que apresentou mais risco à infecção. Frisa-se a importância de avanço no desenvolvimento de pesquisas, a fim de sistematizar informações acerca dos casos de infecção primária e recorrente nesse grupo populacional para melhor compreensão do fenômeno e adequação dos processos de vigilância, medidas de prevenção, controle e atenção a essa categoria profissional.

Foi fator limitante deste estudo a considerável redução do número da amostra de profissionais de enfermagem recrutados da primeira coorte que antecedeu este estudo, com a perda de 335 profissionais. Isso pode ter ocorrido devido à dificuldade de continuação do contato com esses profissionais via smartphone, possivelmente relacionada à rotina de sobrecarga de trabalho que é comum ocorrer na categoria, o que pode deixá-los menos confiantes/dispostos a dar continuidade à pesquisa. Lamentou-se a considerável perda, pois esta dificultou a obtenção de resultados ainda mais relevantes para o contexto atual.

Outra limitação foi o viés de recordação que pode ter ocorrido durante as entrevistas. Em vista principalmente do contexto desafiador da pandemia, há probabilidade de alguns dos profissionais recrutados apresentarem dificuldades em fornecer informações mais precisas durante a abordagem. No entanto, esse viés foi minimizado, visto que boa parte dos profissionais forneceu imagens dos registros vacinais.

Também foi fator limitador deste estudo a escolha de técnica de amostragem não probabilística como abordagem de amostragem. A técnica Respondent-Driven Sampling foi utilizada devido às condições específicas do contexto da pandemia: as restrições necessárias na condução de entrevistas presenciais em vista do lockdown e a falta de registro do quadro de profissionais de saúde atuantes na linha de frente que atendem em salas de emergência, hospitais e novos hospitais de campanha. Ressalta-se a importância da utilização de técnicas amostragem mais relevantes nos contextos que são favoráveis.

Vale salientar também acerca da probabilidade de haver quadros de persistência viral entre as reinfecções encontradas (30). No entanto, essa condição é minimizada pelo fato de ter sido considerada nova infecção após 90 dias da primeira infecção neste estudo.

## Data Availability

O banco de dados e os códigos de análise utilizados na pesquisa estão disponíveis em: https://1drv.ms/f/s!AvWBrOBm0qnih5M4Wf3ModiwMav4YQ?e=bHye54.
